# Family planning and resilience: associations found in a Population, Health, and Environment (PHE) project in Western Tanzania

**DOI:** 10.1007/s11111-018-0310-x

**Published:** 2018-11-29

**Authors:** Karen Hardee, Kristen P. Patterson, Anika Schenck-Fontaine, Sebastiaan Hess, Craig Leisher, Clive Mutunga, Cheryl Margoluis, Cara Honzak

**Affiliations:** 1 Hardee Associates, 5330 26th St N, Arlington, VA 22207 USA; 2 Population Reference Bureau, Washington, DC USA; 3 Leibniz Institute for Educational Trajectories, Bamberg, Germany; 4 Hess Environmental Economic Analyst, Maryculter, UK; 5 The Nature Conservancy, Arlington, VA USA; 6 USAID, Washington, DC USA; 7 Pathfinder International, Watertown, MA USA

**Keywords:** Resilience, PHE, Family Planning, Climate change, Tanzania

## Abstract

Using 2016 household survey data from Tanzania, we define and measure resilience within the context of Population, Health, and Environment programming and quantify the link between resilience and family planning. We created a multicomponent model using confirmatory factor analysis in a structural equation modeling context. Factor loadings for eight defined latent factors of resilience were statistically significant (*p* < 0.001). We created a factor called “FP-MCH” reflecting awareness, attitudes, and access to family planning (FP) and health care services and use of maternal and child health care (MCH) facilities. Analysis, with controls, shows that a 1 standard deviation (SD) increase in FP/MCH was associated with a 0.68 SD increase in resilience (*p* < 0.01), suggesting that the association between FP/MCH and resilience is robust across a range of factors. Analyses showed that the association between FP/MCH is broadly related to the construct of resilience and not through any single component. This study supports the importance of including FP/MCH as part of integrated projects to enhance resilience.

## Introduction

Interest in enhancing the resilience of individuals, households, and communities to contend with climate change-induced and other shocks and stressors in lower- and middle-income countries is growing (Mancini and Bowen 2009; Field et al. 2014; Meyers and Hardee 2017), as is interest in measuring resilience (Quinlan 2014). Resilience has a range of definitions and operates at different scales, but it is generally understood as the ability of an individual, household, community, or system to cope with shocks and stressors, which can be environmental, by responding in ways that maintain their essential functions while expanding their capacity to adapt to change (Folke et al. 2010; Field et al. 2014; UNISDR 2007; Walker et al. 2004). Social and ecological resilience are distinct, but closely linked in literature, particularly in rural areas where communities are reliant upon natural resources for their livelihoods (Adger 2000; Folke et al. 2010).

There is a growing literature on what resilience looks like in practice. In social systems, networks between friends and family, community organization members, and citizens and institutions help build resilience (Adger 2000; Aldrich and Meyer 2014). Resilient ecological systems contain a diversity of flora and fauna that can adapt to environmental changes (Adger 2000; Gunderson 2000). In social–ecological systems, resilience-building practices include adaptive governance (Karpouzoglou et al. 2016), ecosystem management (Virapongse et al. 2016), and disaster risk management (Collier et al. 2009). When social and ecological systems are resilient, they can more readily recover from environmental change and shocks, avoid recurring crises, and develop sustainably (Adger 2000).

Increased attention to the concept of resilience has inspired research into how to best build people’s resilience and ascertain which factors contribute to enhancing resilience. Existing frameworks of resilience have largely ignored the role of population dynamics and the potential for family planning and better reproductive health care to contribute to resilience (Malone 2009; Bremner et al. 2015; Bietsch et al. 2016; Meyers and Hardee 2017; Sathar et al. 2018). However, one exception is USAID’s Program Guidance for Resilience, which states that meeting the unmet need for family planning may be a necessary part of a larger strategy to build resilience (USAID 2012). Furthermore, family planning has been mentioned as a key component in reaching sustainable development goals (Starbird et al. 2016) and as a factor that works on multiple levels to shift people’s ability to adapt to crises, including environmental and climate changes (Smith and Woodward 2014; Husain et al. 2016; Bremner et al. 2015).

While research indicates that integrating family planning and improved reproductive health care into sectors known to contribute to resilience, such as natural resources management, livelihoods, food security, nutrition, and water resources, could further increase that resilience (De Souza 2014; Crist et al. 2017; Naik and Smith 2015; Smith and Smith 2015; Patterson 2018), establishing and measuring the pathways through which resilience is enhanced and the links, if any, among various sectors, is a challenge (Berkes et al. 2003). Currently, the two main links between resilience and family planning and improved reproductive health care found in the literature are (1) the positive effects of the ability to space and plan births on the health of mothers and children (Kock and Prost 2017) and (2) the association between fertility and environmental change. The former fits within the wider link between health and resilience, which posits that good health at the population and individual level are important for short-term response to and long-term recovery from disturbance (NBSB 2014), and that healthy individuals are better able to physically and psychologically withstand events like natural disasters and emotionally cope with the trauma that accompanies any sort of major disturbance (Werner 1995). For the second link between fertility and environmental change, the evidence is mixed, with some studies showing that reduced fertility is related to environmental change, while others show the opposite (Sasson and Weinreb 2017; Brauner-Otto and Axinn 2017; Seller and Gray 2018). More research is therefore needed to more fully understand the link between family planning, reproductive health care, and resilience (PRB and Worldwatch 2014; Yavinsky et al. 2015; Bietsch et al. 2016; Peterson and Giles 2016; Garenne 2017; Grace 2017).

Community-based development projects that simultaneously address livelihoods, natural resources management and conservation, primary health, and reproductive health―commonly known as Population, Health, and Environment or PHE projects―provide an opportunity to study how to measure multiple dimensions of social and environmental resilience and their links with family planning and reproductive health care (Robson et al. 2017). A 2015 synthesis of 35 PHE projects recommended that such projects conduct research to determine whether and how PHE and other integrated projects may contribute to building resilience at the household and community level (Yavinsky et al. 2015).

This research is the first effort to do just that. The paper identifies components of resilience that could be measured in PHE and other integrated development projects, and uses data from a PHE project in western Tanzania to measure resilience and better understand the links between resilience and family planning. The research aims to establish which factors contribute to resilience in the project area, with the ultimate goal of understanding how to build potential resilience among people in ecologically rich regions who rely on natural resources for their livelihoods.

## Case study description: the Tuungane Project

The Nature Conservancy and Pathfinder International have implemented the Tuungane Project since 2012 in collaboration with the Tanzania National Parks Authority (TANAPA), local government authorities, and rural communities. The project aims to improve the health of people, forests, and fisheries in the Greater Mahale Ecosystem through strengthening local governance, improving access to social services (particularly health services), and promoting more sustainable use of natural resources (TNC n.d.).

The Tuungane Project was selected for this data collection and research because the social and environmental challenges facing the local communities provide a good case study in which to examine resilience, and because relevant questions could be added to the project’s midterm survey. The area is remarkable for its rich biodiversity, including 250 endemic species of fish (Coulter 1994) and more than 90% of Tanzania’s endangered chimpanzees (Piel et al. 2013). The communities in the project are extremely remote and therefore have very limited access to physical and social infrastructure. They are heavily reliant on their surrounding natural resources for nearly all of their needs. Access to major markets is poor due to a lack of roads, and the nearest major market (Kigoma) is 20 to 30 h away by ferryboat, depending on weather conditions on the lake (Hess and Leisher 2011). The few health facilities must serve large numbers of people, yet are often far from communities and severely undersupplied and understaffed.

A baseline assessment found that the under-five mortality rate in Mahale was among the 20 highest in the world (Hess and Leisher 2011). Virtually all (95%) of the households were reliant on farming, yet frequently face drought conditions, which contribute to food security issues. Income from fishing, which is the main source of cash income in the area, is threatened by overfishing, especially in fish breeding areas close to shore. Fishermen also faced resource supply challenges. Fully 80% of fishers said the average catch had declined compared to five years prior. Findings from a 2011 assessment of health facilities showed an urgent need to address the primary health care needs of people living in the villages around Mahale Mountains National Park (Pathfinder 2011). Infrastructure, staffing, and medical supplies were of concern in all facilities; none had running water or electricity. In addition, a number of barriers prevented access to health services: transportation, spousal permission to seek care, availability of staff at health facilities, quality of services, cost of services, and the ability to access necessary health-related commodities (Pathfinder International 2011).

Climate change-induced stress is expected from temperature increase and changes in rainfall patterns: climate change models suggest the average temperature in western Tanzania has been warming at a rate of 0.12 °C per decade since the 1950s, and will increase by 1.3 to 2.2 °C over the next 40 years; precipitation will increase slightly, with more rainfall during the rainy season but less during the dry season (Gray 2010). Rainfall is also projected to be less predictable (Gray 2010; Girvetz et al. 2014). In short, the vulnerability of residents in the Tuungane Project areas is multidimensional, encompassing factors such as poor health and nutrition status, poverty, illiteracy, gender inequality, degraded natural resources and low agricultural productivity, and governance failures―all of which will be exacerbated by changes in the climate.

Using household survey data from the Tuungane Project’s 2016 midterm review, this paper fills two gaps in the literature. First, we provide a new approach to identify and measure the components of resilience relevant for PHE programming within a structural equation model framework. With this we aim to show the possibility of more broadly incorporating the measurement of resilience into surveys associated with PHE and other integrated development programming. Second, we provide new evidence of the association between resilience and family planning combined with the use of maternal and child health care facilities, which has been theorized in the literature as important for policy and programming, but for which there is a dearth of evidence.

## Methods

### Identifying components of resilience relevant for PHE and integrated development programming

To understand better which components contribute to building resilience in the context of PHE programming, we conducted an extensive literature review and interviewed PHE practitioners (Meyers and Hardee 2017; PRB 2016). Based on these reviews and a 2016 expert meeting held in Arusha, Tanzania, we identified a number of broad components of resilience for which questions can be added to quantitative and qualitative surveys conducted as part of PHE projects. We sought to quantify resilience to shocks that households in economically and geographically isolated regions adjacent to protected areas typically face and often have to resolve on their own: health crises (e.g., cholera outbreaks), food insecurity, and natural resources management within the context of climate change. Because this research was linked to the Tuungane Project’s planned 2016 household survey, we focused on indicators of resilience that can be measured at the family or household level. We were interested in how people cope with environmental change. Eight initial components and their hypothesized linkages to resilience in the context of integrated PHE programming were social capital/social cohesion (engagement, participation, trust, and support); natural resources management (protecting and rebuilding resources); livelihoods and food security (multiple income sources, caloric intake, food reserves); water, sanitation, and hygiene (WASH) (public health infrastructure); health (physical ability to respond to a crisis); education (information access and processing); knowledge of and experience with climate change (increasing adaptive capacity); and attitudes toward population growth (perceived population pressure on ecological sustainability and community wellbeing).

Based on the literature, we hypothesized that there would be a positive link between resilience and family planning plus access to reproductive health care.

### Survey and sample

Data come from a 2016 cross-sectional survey of 1010 households in 16 villages near the Mahale Mountains National Park in western Tanzania along the coastline of Lake Tanganyika (Hess et al. 2017). The 16 villages formed the survey sample frame, and the sample division over these villages was proportional to the village size (number of households). Tanzanian villages are administratively divided into sub-villages, and for logistical and cost reasons, two sub-villages were selected per village (each village had between three and eight sub-villages). If a village directly bordered Lake Tanganyika, one lake side and one inland sub-village were selected, with the village sample divided over the selected sub-villages proportional to the combined population size of all coastal and inland sub-villages in that village. Complete household lists of the selected sub-villages were drawn up from which households were randomly selected.

For the purpose of this research, a household was defined as a group of people who live together and usually share their food. The average household size was 6.2 people, and 17% of households were female headed. Children under age 15 comprised 52% of the household population in the sample. A majority of household heads (60%) completed primary school or more, while nearly a quarter (22%) of household heads did not have any formal education.

Interviews were conducted using computer-aided personal interviewing (CAPI). The survey instrument included an overall household section and a reproductive health section. The former was answered by one adult household member, preferably the household head, with sufficient knowledge about the household. Nearly half the respondents to this section were male (49%); at the time of the interview, they were 39 years old on average; respondents were the household head (in 60% of the interviews), his or her spouse (31%), a child of the household head (6%), or another household member (3%). The reproductive health section was answered by one female household member between 18 and 49 years old, if present in the household and available. The reproductive health section was completed in 767 households (76% of the sample); respondents were the spouse of the household head (74%), the household head herself (9%), a child of the household head (10%), or another household member (7%). Sometimes, the same respondent answered both sections of the questionnaire. Most of the households without reproductive health section interviews had no eligible female household members (61%). For the remainder, the reasons for not conducting the section interview were not documented. Because refusal rates for the overall interview were very low (1%), it is assumed that refusal was rare and the unavailability of eligible female household members was the primary reason. As much as possible, the interviews were conducted privately to avoid bias, especially the reproductive health section. Enumerators recorded that 95% of these section interviews were not overheard.

### Measures

Using the components of resilience identified above as a guide, and with all of the components in the analysis relating to those theorized prior to the survey, in this section, we describe the variables used to measure the different components of resilience, as well as variables used to measure family planning. For consistency, we use the headings of the final components of resilience in the model described below. Differences between the theorized and final components are explained below and in the findings section. The modeling was an iterative process in which different variables and components were tested. All examined variables and components that were tested are described in this section; Table 1 provides descriptive statistics for all variables used in this study. Details on coding for all items are shown in Appendix Table 4. The estimation of the components is described below.

**Table 1:** Summary statistics

Factor	Item	*N*	Mean	95% CI
Demographic characteristics and covariates	HHH male (%)	1010	83.1	80.6–85.3
	HHH age	977	44.2	43.3–45.1
	HHH completed at least primary school (%)	968	83.6	81.1–85.9
	Anyone in HH employed/self-employed (%)	1010	31.5	28.6–34.5
	Anyone in HH fishes (%)	1010	19.9	17.5–22.5
	Northern village (%)	1010	63.1	60.0–66.1
	Model HH (%)	1010	10.1	8.5–12.3
	HH size	1010	6.2	6.0–6.4
Social cohesion–participation	Influence on village (%)	1010	35.8	32.9–38.9
	TANAPA positive relationship (%)	1010	40.3	37.3–43.4
	Organization member (%)	1010	16.1	13.9–18.6
	Attended public meeting (%)	1010	35.9	33.0–39.0
	Member of BMU (%)	1010	12.1	10.1–14.3
Social cohesion–trust	Trust people in my village (%)	1010	36.2	33.3–39.3
	Trust people in other villages (%)	1010	17.8	15.5–20.3
	Trust village government (%)	1010	44.2	41.1–47.3
Natural resource protection attitudes	Forests should be conserved (%)	1010	88.1	86.0–90.1
	Wildlife should be conserved (%)	1010	88.4	86.3–90.3
	National park should be conserved (%)	1010	89.3	87.2–91.1
	Deforestation causes siltation (%)	1010	68.3	65.3–71.2
	Siltation harms fish (%)	1010	35.5	32.6–38.6
	National park benefits community (%)	1010	54.2	51.1–57.3
	Forest is sufficient for daily needs (%)	1010	43.9	40.8–47.0
Food security and livelihoods and assets	Meet daily needs (%)	1010	60.1	57.6–63.7
	Food shortages (%)	1010	42.3	39.2–45.4
	Food Consumption Score (range: 0–112)	1008	51.8	51.1–52.6
	Low dietary diversity (%)	1008	39.9	36.8–43.0
	Number of crops	1010	3.6	3.5–3.7
	Number of livestock	1010	6.3	6.1–6.4
	Size of farm/forest land (per HH member)	809	1.5	1.4–1.6
	Assets index	1010	0.0	−0.06–0.06
	# income sources	1010	1.7	1.6–1.7
Water, sanitation, and hygiene	Safe water source, dry season (%)	1010	67.6	64.6–70.5
	Safe water source, wet season (%)	1010	72.4	69.5–75.1
	Improved toilet (%)	1010	18.8	16.4–21.4
	Water and soap/ash/sand (%)	1010	46.4	43.3–49.6
Adult use of mosquito bed nets* (%)		765	88.9	86.4–91.0
Women’s highest education level		977	1.6	1.5–1.6
Climate change awareness	Heard of CC (%)	1010	26.7	24.0–29.6
	Observed changes in weather (%)	1010	55.3	52.2–58.4
	CC will have negative effect (%)	1010	33.3	30.4–36.3
Behavior change due to CC (%)		642	12.8	10.3–15.6
Family planning and access to MCH care	Know about FP (%)	1009	63.4	60.4–66.4
	Approve of FP (%)†	1009	74.5	71.7–77.2
	Used FP (%)‡	678	41.4	30.9–52.4
	Better access to FP and health services (%)	1010	55.9	52.8–59.0
	Unmet family planning need (%)	611	51.1	47.0–55.1
	Visited health facility (%)	765	72.2	68.8–75.3
	Home health visit (%)	765	10.2	8.1–12.6
	# of children	706	4.1	4.0–4.3
	Birth spacing (months)	345	30.3	29.0–31.7
	Want more children (%)	1009	69.5	66.5–72.3

*FP* family planning, *MCH* maternal and child health

*Adult use of mosquito bed netting is a proxy for health behavior

†All respondents were given a definition of family planning between the familiarity and the approval questions. This explains how approval can be higher than familiarity

‡The reason family planning use is lower than approval of family planning in part is due to a high desired fertility. The average ideal number of children is high for both women and men (7.4 and 8.4, respectively), and post-survey qualitative research showed approval of family planning relates more to the health benefits for mother and child caused by better spacing, than to the possibility of having smaller families

#### Components of resilience

##### Social cohesion

We included two distinct dimensions of social cohesion: participation and trust. To measure the participation dimension of social cohesion (SC-P), five questions were used. Respondents were asked about their perceived *influence on village government decisions*; whether people in their village have a *positive relationship with TANAPA*; whether any household member was *part of a village organization*; whether any member had *attended a public meeting* about village land-use planning, health issues, lake management, or forest management in the last year; and whether any household member *participated in a Beach Management Unit* (BMU), a community governance body that manages the local fisheries.

To measure the trust dimension of social cohesion (SC-T), we used three questions about whether *people in the respondent’s village can be trusted*, whether *people in other villages can be trusted*, and whether *local government can be trusted*.

##### Natural resource protection attitudes

Seven items were used to measure respondents’ attitudes toward natural resource protection (NR), a proxy for natural resources management. Measuring natural resource management practices directly in a household survey can be problematic given the propensity for social desirability bias to become a factor. While differences can exist between people’s attitudes and practices, we expect changes in attitudes to be reflected in changes in practices over time. Respondents were asked whether *forests should be conserved*, *wildlife should be conserved*, and *the Mahale Mountains National Park should be conserved*. Respondents were also asked whether they believe *deforestation causes siltation*, whether they believe that *siltation harms fish*, whether the *national park provides benefits to the community*, and whether they believe that *there is sufficient forest close by to meet day-to-day needs*.

##### Food security and livelihoods and assets

Households’ level of food security and their access to livelihoods and assets (FLA) were measured using nine items, grouped into two dimensions. To measure the food security dimension of this construct, respondents were asked whether they can *meet their daily needs* and whether they had experienced any *food shortages* in the last 12 months. The World Food Program *Food Consumption Score (FCS)* was calculated using questions on consumption frequency in the previous week of 15 different food categories, collapsed into eight food groups (WFP 2009). Additionally, households’ *dietary diversity* was assessed based on the same questions. Households who had consumed food in four or fewer groups were considered to have low dietary diversity.

To measure the livelihoods and assets dimension of this construct, respondents were asked about the *number of crops* (varieties) they cultivated, the *number of livestock animals* they kept, and the *size of their owned or rented farm or forest land*. Respondents were also asked whether their household had the following items: a bed or mattress, an iron, a sofa, a clock, a radio, a television, a mobile phone, a refrigerator, electricity, solar panels, a generator, a bicycle, a boat without an engine, a boat with an engine, a motorcycle, and a car. This information was used to calculate an *assets index* similar to the DHS Wealth Index (Rutstein and Johnson 2004) by using principal components analysis to create indicator weights, multiplying each standardized indicator (i.e., *z*-score) with the corresponding weight, and then summing across weighted indicators. Finally, respondents were asked how many *sources of income* (i.e., all activities providing food or cash income, including pensions/remittances) their household had.

##### Water, sanitation, and hygiene

To measure a household’s access to safe water, sanitation, and hygiene (WASH), four items were used. To measure whether households have a *safe water source during the dry season*, two questions were combined: Respondents were asked to provide information about whether they used an improved source of drinking water during the dry season (i.e., water from protected wells, rain water, bottled water, water from a vendor, or water from a tanker truck) and whether they treated their water supply to make it safer to drink. The same question was asked about a *safe water source during the wet season*. Respondents were asked whether their household regularly uses an *improved toilet facility*, which was defined as a toilet facility that is not shared and consists of either a pit latrine with a concrete slab or a pour/flush latrine, and were asked to describe their *handwashing practices* and show their handwashing facilities (if any).

##### Health behavior

Health behavior, which has been identified as an important factor of resilience (NBSB 2014), was measured through a proxy, adult use of mosquito bed nets. Female respondents of the reproductive health section were asked whether they slept under a mosquito net the night before the interview. Other health variables that were considered as additional proxies for health behavior include general disease prevalence, malaria incidence, and measles immunization for children. However, general disease prevalence did not correlate highly with any other health variable, and neither malaria incidence nor measles immunization for children had enough variation to sufficiently discriminate between groups and therefore could not be used in the analysis. Although our health component may not capture the full importance of health behavior within resilience, we elected to include the above proxy as we did not want our model to imply that health behavior is not important for resilience.

##### Women’s highest education level

Education has been positively linked with resilience (Van der Land and Hummel 2013; Lutz and Striessnig 2015). As a proxy measure of the level of education of the members in a household, the highest level of education for the most highly educated woman in each household was included in the model (0 = *none or less than primary*, 1 = *some primary*, 2 = *completed primary*, 3 = *some secondary*, 4 = *completed secondary*, 5 = *higher than secondary*). Additionally, we considered including the education level of the household head, the highest education level of any member, and the average education level of all household members. However, because women’s education is closely linked with economic development and family and child wellbeing, the education level of the most highly educated woman in the household was deemed to be more theoretically relevant.

##### Climate change awareness

Climate change awareness (CC-A) was measured using four items. Respondents were asked whether they had *heard of the term “climate change”* (*mabadiliko tabia nchi* in Kiswahili). Respondents were also asked whether they had *observed changes in the weather since they were young*, whether they believed that *climate change would affect their household*, and whether they had *changed how they work or live* because of these observed changes.

#### Family planning and access to maternal and child health care

The construct family planning and access to maternal and child health care (FP-MCH) was measured using ten questions; the questions about access to health care were included in the reproductive health section of the questionnaire and were mainly intended to measure access to maternal and child care; thus, they were grouped with family planning knowledge and uptake. This factor included both family planning and maternal and child health care because health and development programming commonly integrates these two services. Respondents were asked whether they *know what family planning is*, whether they *approve of using family planning*, whether their household had *better access to family planning and health care services* compared to 5 years ago, and whether they currently used any method of family planning. *Unmet need for family planning* is a composite indicator and was determined through a series of questions in the reproductive health section, e.g., the desire to avoid pregnancy, the use of contraceptives, marital status, and fecundity. The indicator was calculated following Bradley et al. (2012). Respondents of the reproductive health section also provided information about whether they *visited a health care facility* and whether they *received a home visit from a health care worker* in the 12 months prior to the survey. Community health workers visit each household to support general maternal and child health and family planning needs. Finally, respondents were asked about the *number of children* they had, the *spacing between their children’s births*, and whether they wished to *have more children*. In the initial inventory of potential indicator variables, we considered the variable *peoples’ perceptions of population increase as problematic* as a variable related to resilience in that such perceptions might be related to family planning; however, this variable was eliminated in favor of more direct attention to family planning and access to maternal and child health services.

#### Covariates

Finally, we also included a set of control variables. Demographic controls included the sex and age of the household head and the household size. Socioeconomic controls included whether anyone in the household was employed or ran a business, and whether fishing was a source of household income. Also included was whether the household was a “model household,” which is one of the Tuungane project interventions. Model households are volunteer households that receive training around health, hygiene, the environment, and the relationship between these issues, and then serve as peer educators in their community. Finally, whether a household’s village was located in the project’s northern or southern areas was also included as a control variable to capture differences in remoteness, ethnic makeup, and livelihoods of the areas.

### Analytic strategy

We used confirmatory factor analysis (CFA) to measure the hypothesized components of resilience, as well as resilience itself. CFA is a theory-driven analysis of the relationships between observed variables that tests whether the covariance matrix of the observed data matches the covariance matrix of a hypothesized model (Schreiber et al. 2006). Using this approach, it is possible to estimate exogenous latent constructs that are shared by a set of observed variables, which are considered endogenous or determined by the latent construct. In a structural equation modeling framework (SEM), CFA is also called a measurement model. Besides measurement models, SEM also allows the estimation of structural models, which estimate relationships between latent and observed variables using regression. The goal of measurement and structural models in SEM is to minimize the difference between the covariance matrix of the hypothesized model and the actual covariance matrix of the observed data. In addition to allowing us to model the complex relationships hypothesized in our model, SEM also allows us to estimate unbiased coefficients despite potential multicollinearity (see Appendix Table 5 for the correlation matrix). SEM models require sufficient sample size, with a minimum of 10 observations for each estimated parameter (Schreiber et al. 2006). In our most complex model, we estimate 74 parameters. With a sample size of 1010, we have a ratio of observations to parameters of 13.7:1.

We followed a two-step process to develop a comprehensive measure of resilience using CFA. First, we estimated first-order measurement models for each multi-item construct that was hypothesized to be a component of resilience (i.e., *social cohesion–participation; natural resource protection attitudes; food security and livelihoods and assets; water, sanitation, and hygiene; and climate change awareness*). We repeated the same process to create a measurement model of *family planning and access to MCH care*. Second, we estimated a second-order measurement model that incorporates the aforementioned first-order measurement models of the components of resilience, as well as three observed variables (i.e., adult use of mosquito bed nets, women’s highest education level, and changed behavior due to climate change).

Once satisfactory measurement models had been fitted, we used SEM to examine the association between *resilience* and *family planning and access to MCH care*. We also tested the hypothesized structural model with the inclusion of all covariates. Finally, we examined the direct association between *family planning and access to MCH care* and each individual component of resilience to assess whether an association with a particular component of resilience is driving the overall relationship.

All measurement and structural models were estimated using Mplus 6.1 (Muthén and Muthén 2012) with maximum likelihood estimation using weighted least squares estimators using a diagonal matrix with robust standard errors and the Satorra–Bentler chi-squared test for model fit. This estimation approach is most appropriate for models that include binary or ordered categorical dependent variables, as well as non-normally distributed continuous data, as is the case in our models (Brown 2006; Savalei 2014). Pairwise deletion was used to address missing data, which uses information from any observation with available data for both variables in a pair to estimate the correlation matrix. While pairwise deletion results in different numbers of observations being used for different parts of a model, this approach maximizes the data available. This approach is adequate when data are missing at random (MAR), that is, when missingness is explained by observed variables (Allison 2001). Given that the vast majority of missingness is related to whether households participated in the reproductive health section, this is a reasonable assumption.

For all measurement and structural models, model fit was evaluated using a combination of three model fit statistics, since each goodness-of-fit statistic operates on different assumptions (Hoyle and Panter 1995). The comparative fit index (CFI; Gentler 1990) ranges from 0 to 1, with 1 indicating a perfectly fitted model. Values of 0.9 or higher signify good model fit (Bollen 1989; Hoyle and Panter 1995). The root mean square error of approximation (RMSEA) was also used in this study, and values below 0.05 are considered evidence of good model fit (Browne and Dudek 1993). Also presented here is the *χ*^2^ statistic; a non-significant value indicates good model fit. However, the *χ*^2^ statistic is sensitive to sample size and the large sample size of this study renders this statistic largely undiagnostic. Therefore, the *χ*^2^ ratio (*χ*^2^/df) is also presented, as this adjusts for sample size. Good fit is indicated by a *χ*^2^ ratio between 1 and 3 (Arbuckle and Wothke 1999).

## Results

### Measurement models establishing proposed latent factors

We composed all first-order latent factors, including *family planning and access to MCH care*, from their underlying indicator variables through an iterative process. The final unadjusted results for the first-order measurement models are presented in Table 2.

**Table 2:** Summary of confirmatory factor analysis measurement models

	Unstandardized coefficient	Standardized coefficient
Measurement model 1: social cohesion–participation (SC–P)		
SC–P → influence on village government decisions	1.00^1^	0.49
SC–P → positive relationship with TANAPA	1.05***	0.51
SC–P → membership in village organization	1.57***	0.77
SC–P → attended public meeting	1.12***	0.55
SC–P → participated in BMU	1.21***	0.59
*Model fit:* CFI = 0.967, RMSEA = 0.049, *χ*^2^ (df) = 17.048 (5), *χ*^2^ ratio = 3.410		
Measurement model 2: social cohesion–trust (SC–T)		
SC–T → trust people in my village	1.00^1^	0.87
SC–P → trust people in other villages	0.49***	0.42
SC–P → trust village government	0.61***	0.53
*Model fit:* CFI = 1.000 RMSEA = 0.000, *χ*^2^ (df) = 0.000 (0), *χ*^2^ ratio = n/a		
Measurement model 3: natural resource protection attitudes (NR)		
NR → forests should be conserved	1.00^1^	0.75
NR → wildlife should be conserved	1.21***	0.91
NR → Mahale National Park should be conserved	1.18***	0.88
NR → deforestation causes siltation	0.80***	0.60
NR → siltation harms fish	0.83***	0.62
NR → national park provides benefits to community	0.83***	0.62
*Model fit:* CFI = 0.992 RMSEA = 0.042, *χ*^2^ (df) = 22.412 (8), *χ*^2^ ratio = 2.802		
Measurement model 4: food security and livelihoods and assets (FLA)		
FA → can meet daily needs	1.00^1^	0.55
FA → no food shortages	1.02***	0.56
FA → food consumption score (FCS)	9.49***	0.43
FA → number of crops	1.43***	0.41
FA → number of livestock animals	8.93***	0.45
FA → assets index	0.75***	0.41
*Model fit:* CFI = 0.948, RMSEA = 0.059, *χ*^2^ (df) = 40.182 (9), *χ*^2^ ratio = 4.465		
Measurement model 5: water, sanitation, and hygiene (WASH)		
WH → safe water source during dry season	1.00^1^	0.53
WH → improved toilet facility	1.02***	0.54
WH → water and soap/ash/sand to wash hands	0.88***	0.47
*Model fit:* CFI = 1.000 RMSEA = 0.000, *χ*^2^ (df) = 0.000 (0), *χ*^2^ ratio = n/a		
Measurement model 6: climate change awareness (CC-A)		
CC → heard the term “climate change”	1.00^1^	0.63
CC → observed changes in weather since young	1.28***	0.81
CC → climate change would negatively affect household	0.97***	0.61
*Model fit:* CFI = 1.000, RMSEA = 0.000, *χ*^2^ (df) = 0.000 (0), *χ*^2^ ratio = n/a		
Measurement model 7: family planning and access to MCH care (FP-MCH)		
FP → know what family planning is	1.00^1^	0.67
FP → approve of family planning	0.58***	0.40
FP → better access to family planning and health care services	0.84***	0.58
FP → visited a health care facility	0.56***	0.40
FP → received home visit from health care worker	0.59***	0.41
*Model fit:* CFI = 0.994, RMSEA = 0.015, *χ*^2^ (df) = 6.162 (2), *χ*^2^ ratio = 3.076		
Measurement model 8: resilience		
Resilience → SC	1.00^1^	0.82
Resilience → NR	1.33***	0.73
Resilience → CC	1.26***	0.66
Resilience → WH	0.48***	0.50
Resilience → FA	0.49***	0.41
Resilience → adult use of mosquito bed nets	1.00^1^	0.42
Resilience → behavior change due to climate change	0.98***	0.41
Resilience → highest women’s education level	0.87***	0.34
*Model fit:* CFI = 0.907, RMSEA = 0.036, *χ*^2^ (df) = 675.678 (294), *χ*^2^ ratio = 2.298		

Sample size for each model is 1010

*CFI* comparative fit index, *RMSEA* root mean square error of approximation, *χ*^*2*^ *ratio χ*^2^/df

^1^According to requirements for SEM analyses, one variable loading on each latent factor was set equal to 1.00 to set the metric for that factor. As a result, significance values are not calculated for these variable loadings

***p < 0.0001

Unstandardized factor loadings are presented to show significance. Standardized factor loadings are presented, with the variance of the latent factor set to 1.00, so that factor loadings can be compared. All first-order measurement models fit well, with at least two fit indices indicating good fit; all factor loadings of the observed variables on the hypothesized factors were significant. Three latent factors―*social cohesion–trust*; *water, sanitation, and hygiene*; and *climate change awareness*―are perfectly fitted because these two factors include only three observed variables and use all degrees of freedom, which means that the models are as complex as the covariate matrices that they explain.

The latent factor *social cohesion–participation* (SC–P) was indicated by respondents’ perception of their influence on village government decisions, their perception of the village’s relationship with TANAPA, membership in a village organization, attendance at public meetings, and participation in a beach management unit (BMU). Membership in a village organization loaded most highly on this factor (*β* = 0.77, *p* < 0.0001). The latent factor *social cohesion–trust* (SC–T) was indicated by respondents’ trust in people in their village, in people in other villages, and in the village government. Trust in people in respondents’ own village loaded most highly on this factor (*β* = 0.87, *p* < 0.0001). The latent factor *natural resource protection attitudes* (NR) was indicated by a respondents’ opinion of whether forests, wildlife, and the national park should be conserved, whether deforestation causes siltation, whether siltation harms fish, and whether the national park provides community benefits. The opinion that wildlife should be conserved loaded most highly on this factor (*β* = 0.91, *p* < 0.0001). The variable measuring belief about the presence of sufficient and proximate forest for day-to-day needs was excluded from the final measurement model because of a notably low factor loading and poor model fit.

The latent factor food security and livelihoods and assets (FLA) reflects the physical livelihoods factor of resilience and was indicated by a household’s ability to meet daily needs, the lack of food shortages, the food consumption score (FCS), the number of crops and livestock animals, and an assets index. Having no food shortages loaded most highly on this factor (*β* = 0.56, *p* < 0.0001). The two dimensions of this factor were combined into a single latent construct based on the evaluation of measurement model fit, and the need to have at least three variables per factor. Three observed variables were excluded from this model based on very low factor loadings and poor model fit: household dietary diversity, the amount of owned or rented farm or forestland, and number of income sources. The latent factor *water, sanitation, and hygiene* (WASH) was indicated by a household’s access to a safe water source during the dry season, improved toilet facilities, and the use of water and soap, ash, or sand to wash hands. Improved toilet facilities loaded the highest on this factor (*β* = 0.54, *p* < 0.0001). The observed variable measuring access to a safe water source during the wet season was excluded from the final measurement model because of a very low factor loading and poor model fit, and because there was high correlation between access to safe water in the dry and wet seasons.

The latent factor *climate change awareness* (CC-A) was indicated by whether the respondent had heard of the term “climate change,” had observed changes in the weather, and believed that climate change would negatively impact their household. Having observed changes in the weather loaded the highest on this factor (*β* = 0.81, *p* < 0.0001). The observed variable reflecting changed behavior due to observed changes in weather patterns was initially included in this factor. However, due to low factor loadings and poor model fit, it was included as a separate variable in the second-order measurement model.

Finally, the latent factor *family planning and access to MCH care* (FP-MCH) was indicated by respondents’ knowledge of family planning, approval of the use of family planning, access to family planning and health care services, and their use of a health care facility or home visit services, mainly for maternal and child health services. Knowledge about family planning loaded the highest on this factor (*β* = 0.67, *p* < 0.0001). Four observed variables were excluded from the final measurement model due to very low factor loadings and poor model fit: whether respondents had an unmet need for family planning, the number of children respondents have, the time spacing between births, and whether the respondents want to have more children.

All but three of the first-order measurement models were overidentified with degrees of freedom greater than 0. The measurement models for *social cohesion–trust*; *water, sanitation, and hygiene*; and *climate change awareness* were identified with degrees of freedom equal to 0.

Next, we tested our operationalization of resilience as a second-order latent factor (Table 2). All first-order measurement models (i.e., latent factors) are included in the second-order model of resilience and in all structural models below, with the exception of the latent factor *social cohesion–trust*, which was excluded due to poor model fit. Again, standardized factor loadings are presented, with the variance of the latent factor set to 1.00, to enable a comparison of factor loadings. This second-order measurement model fit well. The CFI, the RMSEA, and the *χ*^2^ ratio all indicated good fit of the second-order measurement model, and all factor loadings of the first-order factors and observed variables on the hypothesized second-order factors were significant. We operationalized resilience as a factor indicated by *social cohesion–participation; natural resource protection attitudes; food security and livelihoods and assets; water, sanitation, and hygiene; climate change awareness;* adult use of mosquito bed nets; women’s highest education level in a household; and reported behavior change due to observed climate change. *Social cohesion*–*participation* loaded most highly on resilience (*β* = 0.82, *p* < 0.0001). This was followed by *natural resource protection attitudes* (*β* = 0.73, *p* < 0.0001), *climate change awareness* (*β* = 0.66, *p* < 0.0001), and *water, sanitation, and hygiene* (*β* = 0.50, *p* < 0.0001). The factor loadings for the other included factors and observed variables were lower but still significant; the factor loadings for adult use of mosquito bed nets (*β* = 0.42, *p* < 0.0001), *food security and livelihoods and assets* (*β* = 0.41, *p* < 0.0001), and observed behavior change due to climate change (*β* = 0.41, *p* < 0.0001) were very similar to each other. The lowest factor loading was for women’s education level (*β* = 0.34, *p* < 0.0001). Including other specifications of the education variable (see “Measures” section) had very similar outcomes. This model was overidentified with degrees of freedom greater than 0.

### Association between resilience and family planning and access to MCH care

**Fig. 1:**
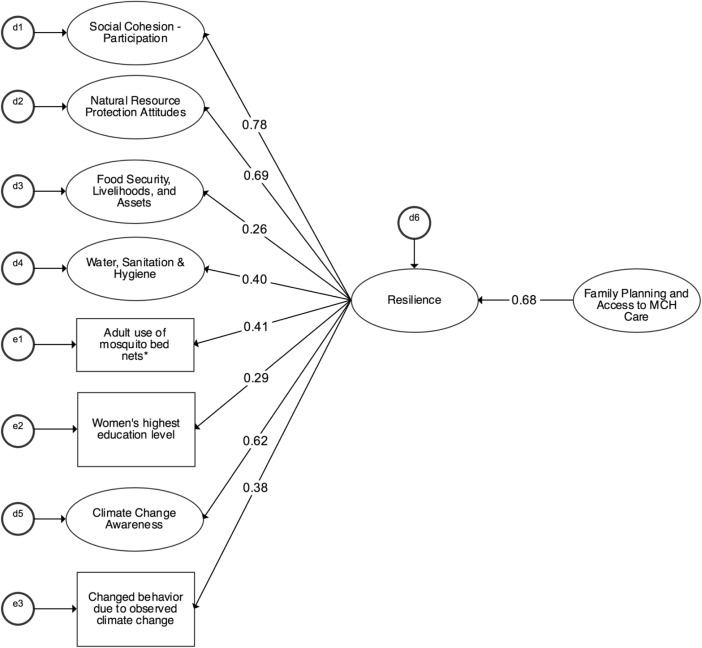
Unadjusted association between resilience and family planning and access to maternal and child health care. *Notes:* Standardized paths are shown; all paths are significant at least at *p* < 0.01. Ovals represent latent factors and rectangles represent observed variables. *N* = 1010. CFI = 0.902, RMSEA = 0.032, *χ*^2^ (df) = 879.077 (428), *χ*^2^ ratio = 2.054. *, adult use of mosquito bed nets is a proxy for health behavior

SEM analysis was again used to test the hypothesized association between *resilience* and *family planning and access to MCH care*. The results of the unadjusted structural model without the inclusion of any covariates are presented in Fig. 1. All factor loadings are standardized, with the variance of the latent factors set to 1.00 to allow comparison across factor loadings. While the factor loadings on resilience differ from the factor loadings presented in the second-order measurement model because of the inclusion of *family planning and access to MCH care* in the structural model, the relationships reflected are substantively the same. The regression coefficient representing the association between *resilience* and *family planning and access to MCH care* is also standardized. This unadjusted model fit well (CFI = 0.902, RMSEA = 0.032, *χ*^2^ (df) = 879.077 (428), *χ*^2^ ratio = 2.054), and all factor loadings were significant. These results suggest that a 1 SD increase in *family planning and access to MCH care* was associated with a 0.67 SD increase in *resilience* (*p* < 0.01). This positive association is large and significant.

**Fig. 2:**
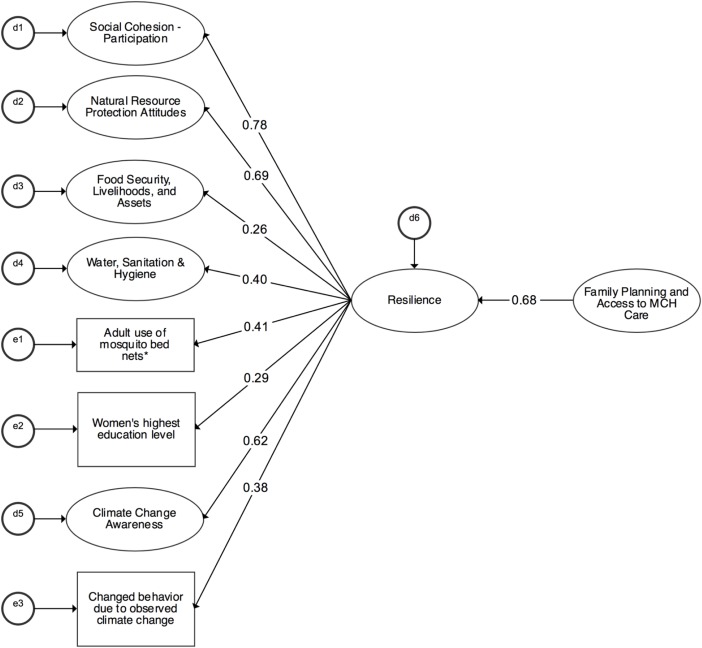
Adjusted association between resilience and family planning and access to maternal and child health care. *Notes:* Standardized paths are shown; all paths are significant at least at *p* < 0.01. *N* = 1010. CFI = 0.865, RMSEA = 0.032, *χ*^2^ (df) = 1090.614 (528), *χ*^2^ = 2.066. Covariates include sex and age of the household head, household size whether anyone in the household was employed or ran a business, whether fishing was a source of household income, whether the household was a “model household,” and the location of the village. Ovals represent latent factors and rectangles represent observed variables. Asterisk, adult use of mosquito bed nets is a proxy for health behavior

Figure 2 shows results from the adjusted structural model, which controlled for the sex and age of the household head, whether anyone in the household was employed or ran a business, whether fishing was a source of household income, whether the household was a model household, and the location of the village. While the inclusion of these covariates reduced the fit of this model slightly, two of the fit indices still indicated a good fit (CFI = 0.861, RMSEA = 0.032, *χ*^2^ (df) = 1198.084 (597), *χ*^2^ ratio = 2.007) and all factor loadings were significant. Moreover, the inclusion of these covariates did not significantly alter the association between *resilience* and *family planning and access to MCH care*. Specifically, the adjusted model results show that a 1 SD increase in *family planning and access to MCH care* was associated with a 0.68 SD increase in *resilience* (*p* < 0.01). This suggests that the association between *resilience* and *family planning and access to MCH care* is robust across a range of individual, household, and village factors.

Finally, it is important to note that being a “model household” was associated with higher levels of *social cohesion–participation* (*β* = 0.17, *p* < 0.001); *water, sanitation, and hygiene* (*β* = 0.11, *p* < 0.05); and *family planning and access to MCH care* (*β* = 0.23, *p* < 0.001). Similarly, being a female-headed household was associated with lower levels of *social cohesion–participation* (*β* = − 0.18, *p* < 0.01); *natural resource protection attitudes* (*β* = − 0.05, *p* < 0.05); *food security and livelihoods and assets* (*β* = − 0.23, *p* < 0.001); and *climate change awareness* (*β* = 0.19, *p* < 0.001). Appendix Table 6 provides the coefficients for all covariates in each of the first-order measurement models. All structural models were overidentified.

### Association between factors of resilience and family planning and access to MCH care

Finally, in order to examine whether the association between *family planning and access to MCH care* and *resilience* is driven largely by an association with a specific component of *resilience*, we also used SEM to estimate the direct associations between the components of *resilience* and *family planning and access to MCH care*. The results of these simultaneous regressions are presented in Table 3, in which regression coefficients are standardized. The model, which included all control variables, fit well (CFI = 0.866, RMSEA = 0.032, *χ*^2^ (df) = 1149.524 (569), *χ*^2^ ratio = 2.020). The results show that *family planning and access to MCH care* was significantly associated with all components of resilience except *food security and livelihoods and assets*. However, *family planning and access to MCH care* was most significantly associated with *social cohesion–participation.* Specifically, a 1 SD increase in *family planning and access to MCH care* was associated with a 0.60 SD increase in *social cohesion–participation* (*p* < 0.01). *Family planning and access to MCH care* was also strongly associated with *climate change awareness* (*β* = 0.42, *p* < 0.01), *natural resource protection attitudes* (*β* = 0.41, *p* < 0.01), and adult use of mosquito bed nets (*β* = 0.40, *p* < 0.01). Though the associations were lower, *family planning and access to MCH care* was also significantly associated with *water, sanitation, and hygiene* (*β* = 0.33, *p* < 0.01); observed behavior change due to climate (*β* = 0.29, *p* < 0.01); and women’s education level (*β* = 0.22, *p* < 0.01).

**Table 3:** Direct association between components of resilience and family planning and access to MCH care

	(1) Social cohesion–participation	(2) Natural resources	(3) Food security and livelihoods and assets	(4) Water, sanitation, and hygiene	(5) Adult use of mosquito bed nets	(6) Women’s highest education level	(7) Climate change awareness	(8) Behavior change due to climate change
Family planning and access to MCH care	0.60** (0.08)	0.41** (0.09)	0.07 (0.04)	0.33** (0.07)	0.40** (0.14)	0.22** (0.08)	0.42** (0.09)	0.29** (0.14)

Coefficients are standardized; robust standard errors in parentheses; sample size for each model is *n* = 1010. Covariates include sex and age of the household head, whether anyone in the household was employed or ran a business, whether fishing was source of household income, whether the household was a “Model Household,” and the location of the village. Fit statistics: CFI = 0.866, RMSEA = 0.032, *χ*^2^ (df) = 1149.524 (569), *χ*^2^ = 2.020

*SC*–*P* social cohesion–participation, *NR* natural resource protection attitudes, *CC-A* climate change awareness, *WASH* water, sanitation, and hygiene, *FLA* food security and livelihoods and assets

**p < 0.01

### Alternative specification of family planning

In order to test whether the association between *resilience* and *family planning and MCH* care remains robust with the exclusion of MCH care access, we replicated the estimated path model with an alternative specification of the family planning factor that does not include access to MCH care. The alternative *family planning* factor was indicated by respondent’s knowledge of family planning (*β* = 0.57, *p* < 0.0001), approval of the use of family planning (*β* = 0.49, *p* < 0.0001), and whether respondents have an unmet family planning need (*β* = − 0.58, *p* < 0.0001). The factor is perfectly fitted because it includes only three observed variables (CFI = 1.000, RMSEA = 0.000, *χ*^2^ (df) = 0.000 (0)). We then used SEM to test the hypothesized association between *resilience* and *family planning*. The results from the adjusted structural model, which included all control variables specified above, show that a 1 SD increase in *family planning* was associated with a 0.71 SD increase in *resilience* (*p* < 0.01) (CFI = 0.865, RMSEA = 0.032, *χ*^2^ (df) = 1090.614 (528), *χ*^2^ ratio = 2.066). This suggests that the association between *resilience* and *family planning* is not solely driven by the inclusion of MCH care access.

## Discussion

This paper presents results of a first-of-its-kind effort to define and measure resilience within the context of PHE multisectoral programming and identify and quantify the link between resilience and family planning combined with use of maternal and child health facilities. The analysis, using data from a 2016 survey in the Tuungane Project in western Tanzania, confirms that resilience can be measured in PHE and other integrated community-based development programming. These measures of resilience can help direct programming. This paper provides evidence that family planning with access to MCH care is positively associated with resilience, a link that had been posited previously but lacked sufficient quantitative evidence.

Based on the literature on resilience and interviews with PHE practitioners, questions related to broad components of resilience were included in the 2016 survey. Among a number of indicators that were hypothesized to comprise resilience, the following multi-item components of resilience emerged: *social cohesion–participation*; *natural resource protection attitudes*; *food security* and *livelihoods and assets*; *water*, *sanitation*, *and hygiene*; and *climate change awareness*. We added an additional three observed variables to the model of resilience: adult use of mosquito bed nets (as a proxy for health behavior), women’s highest education level (as a proxy for education), and changed behavior due to climate change (as an added dimension of climate change). The strength of these factor loadings reflects the relative importance of each of these components to the core construct of resilience. All factor loadings were significant; among these, *social cohesion*–*participation* had the highest factor loading on resilience, followed by *natural resource protection attitudes*, *climate change awareness*, and *water*, *sanitation*, *and hygiene.* This finding makes theoretical sense given that the literature on resilience highlights the primacy of social capital in dealing with adverse weather events, the need for natural resources as a means of rebounding from negative environmental change, and the need for maintaining public health and access to clean water (Meyers and Hardee 2017). While adjustments were made based on available variables from the dataset and on model fit, seven of the eight original components of resilience identified in the literature and in the expert consultation conducted to inform the 2016 survey fit in the final model.

After including *family planning/access to MCH care* in the model, all factor loadings remained significant, and a positive and strong association between *resilience* and *family planning/access to MCH care* was found, both before and after the inclusion of a range of control variables. Moreover, results using an alternative specification of family planning that excludes the maternal and child health care component of this factor confirms that the association between resilience and *family planning/access to MCH care* is not driven by the inclusion of health care access measures. Thus, there is a strong and significant association between resilience and family planning alone.

The results also show that there is an association between *family planning/access to MCH care* and the individual components of resilience, with the exception of *food security and livelihoods and assets*. These results suggest the association between *family planning and access to MCH care* and *resilience* is not driven by an association with only one or two specific components of resilience. That *food security and livelihoods and assets* were not significantly associated with *family planning/access to MCH care* was surprising and needs more exploration, given the strong theoretical link between food availability and number of mouths to feed in a family (Bremner 2012; Smith and Smith 2015; Borwankar and Amieva 2015).We hypothesize that the theoretical family planning and food security/nutrition links are not reflected in our data because our family planning factor does not reflect reduced fertility, which is an important driver in these theoretical pathways, and we did not measure all food security and nutritional aspects (e.g., stunting). Another possible reason for the lack of association could be temporal: that *family planning and access to MCH care* is significantly associated with future, but not concurrent, *food security and livelihoods and assets.* Additional research is necessary to further explore the temporal variations in the relationships between all components of *resilience* and *family planning/access to MCH care*. Attention to better measurement of food security and livelihoods and assets is also warranted.

This study had some limitations. Because it was the first of its kind, the components used to define resilience may not be complete. Furthermore, the questions asked in the survey about the components of resilience were not always satisfactory to measure the constructs in the model. For example, we had to use mosquito bed net use as a proxy for health behavior because other questions asked about health behavior did not perform well in the analysis, including questions about general disease prevalence, malaria incidence, and measles immunization for children. We decided to keep the bed net measure in the model because health is a recognized component of resilience (NBSB, 2014), and we did not want our model to imply that health is not important for resilience. Future surveys should include a wider range of questions on health behavior. We also suggest further work on measuring food security and livelihoods and assets. Additionally, because two of the latent constructs in the final model are measured using only three observed variables, it is possible that these factors are too perfectly reflective of the unique variation and relationships reflected in this particular dataset and that the results of these models may not generalize to other populations. Therefore, it is possible that the associations between resilience and family planning and access to maternal and child health care and, indeed, the nature of resilience may differ in other samples. For that reason, replication of these measures with other data sources and their further refinement is a necessary step for future research.

It is also possible that, because the majority of missing data is due to households not having an eligible respondent for the reproductive health section, the results are biased because of the selectivity of the reproductive health section sample. Only households with eligible respondents who were available provided information for several variables in the model that come from this section (e.g., adult use of mosquito bed nets, use of family planning, and visits to a health facility). These households had a younger head of household and had more children than households that did not participate in the reproductive health section. The women in these households are also more educated on average. Therefore, because our model estimation relies more heavily on these younger and more educated households for the associations and covariances of several family planning variables and for mosquito net use, it is possible that we overestimate the role of education and mosquito net use in resilience and, thus, their association with family planning and maternal health care. Finally, a limitation of SEM models is that they do not allow for causal inference, and the association between family planning and access to maternal and child health care and resilience can reflect a relationship that goes in either direction. Therefore, additional research on the directionality of this relationship is needed.

Given the complexity of the models, there was insufficient power to conduct sub-analyses of the findings by group (e.g., female vs. male heads of household), which would show whether the components of resilience have a different configuration for different sub-groups and whether the association between resilience and family planning and access to MCH care varies among different groups. However, by controlling for a set of demographic, socioeconomic, and other household variables, it was possible to assess which characteristics are likely associated with differential levels in each of the components of resilience. Being a “model household” was associated with higher values on *family planning and access to MCH care*; *social cohesion–participation*; and *water, sanitation, and hygiene*. This suggests that the Tuungane Project’s model households are reflecting the desired behavior changes. Yet, the association between resilience and *family planning and access to MCH care* is significant for all households, including those that did not receive specific training on health, hygiene, and the environment. Additionally, being a female-headed household was associated with lower values of *social cohesion–participation*, *natural resource protection attitudes*, *food security and livelihoods and assets*, and *climate change awareness*, which suggests that female-headed households are likely less resilient on average than male-headed households. Together, these results are suggestive of sub-group differences in resilience; further research is warranted to investigate the extent of these differences. Additional analysis of other demographic factors, including length of time in the village, migration history, and marital status, would be beneficial.

Moreover, this study has only used indicators of resilience that were collected at household level. While some of these in part also reflect community properties, such as the indicators included in the social cohesion component, our measure of resilience cannot fully capture all its facets, as vulnerability to shocks and changes is determined at multiple levels and varies according to local community characteristics or even the quality of national governance. It would be hard for PHE projects to measure resilience at all these levels, but testing how resilience is measured at the household level could be combined with community-level indicators to create a more encompassing measurement of resilience and would be an interesting addition to the research presented here.

Finally, in factor analysis, certain variables are excluded if they do not fit together well in a single factor. Therefore, our constructs do not include all the data collected in the survey. For example, in food security/nutrition, we created two indicators showing partially conflicting results: (1) a food consumption score showing the households overall had good nutritional values and (2) diet diversity indicating a generally low diet diversity in the study area. Only the former of these indicators fit well within the food security and livelihoods and assets factor, and therefore, the factor does not reflect the full nutritional picture of the area because of the required conditions for factor analysis. Thus, the constructs are sometimes narrower than their factor titles might suggest, and when interpreting the results, it is important to keep in mind which variables comprise each factor. We have listed all variables that were looked at in the analysis in Table 2 since some that did not fit the data from the Tuungane survey might perform better with data from other settings.

Acknowledging these limitations, this study represents a start in the identification of resilience indicators that can be incorporated into the project design and measured within PHE projects as well as other integrated multisectoral development projects to measure resilience, with adaptations for particular project settings. The study also provides evidence of the importance of including family planning and access to maternal and child health care among the components of programming to strengthen resilience. Measuring these indicators over time can help assess whether social resilience is increasing in project areas. This information can be analyzed in conjunction with data on natural resources management and climate change, including gradual change and sudden shocks. In addition, this analysis can inform the design and implementation of holistic programs to strengthen resilience. This analysis can also inform the design and implementation of integrated multisectoral programs to strengthen resilience, helping communities meet multiple development needs―including reproductive health―and cope with climate and environmental shocks.

## Appendix

**Table 4:** Variable coding structure

Variables	Coding
Demographic characteristics and covariates	
HHH male (%)	Dummy: 1 = male; 0 = female
HHH age	Continuous
HHH completed primary school (%)	Dummy: 1 = HHH completed primary or higher level of education; 0 = lower level of education
HHH received no formal education (%)	Dummy: 1 = HHH did not at least have some primary education; 0 = at least some primary education
Anyone in HH employed/self-employed (%)	Dummy: 1 = one or more household members are employed/have business; 0 = no one in the household is employed/has a business
Anyone in HH fishes (%)	Dummy: 1 = one or more household members fish for food or cash income; 0 = no one in the household fishes
Northern village (%)	Dummy: 1 = household is in one of the northern villages; 0 = household is in southern village
Model HH (%)	Dummy: 1 = household participates in model household program; 0 = household does not participate
HH size	Continuous
Social cohesion–participation	
Influence on village (%)	Dummy: 1 = have influence; 0 = have no influence/not sure
TANAPA positive relationship (%)	Dummy: 1 = yes; 0 = no/not sure
Organization member (%)	Dummy: 1 = yes; 0 = no
Attended public meeting (%)	Dummy: 1 = yes; 0 = no
Member of BMU (%)	Dummy: 1 = yes; 0 = no
Social cohesion–trust	
Trust people in my village (%)	Dummy: 1 = can always/usually be trusted; 0 = can never/rarely/sometimes be trusted
Trust people in other villages (%)	Dummy: 1 = can always/usually be trusted; 0 = can never/rarely/sometimes be trusted
Trust village government (%)	Dummy: 1 = can always/usually be trusted; 0 = can never/rarely/sometimes be trusted
Natural resource protection attitudes	
Forests should be conserved (%)	Dummy: 1 = yes; 0 = no/not sure/neutral
Wildlife should be conserved (%)	Dummy: 1 = yes; 0 = no/not sure/neutral
National park should be conserved (%)	Dummy: 1 = yes; 0 = no/not sure/neutral
Deforestation causes siltation (%)	Dummy: 1 = yes; 0 = no/not sure/neutral
Siltation harms fish (%)	Dummy: 1 = yes; 0 = no/not sure/neutral
National park benefits community (%)	Dummy: 1 = yes; 0 = no/not sure/neutral
Forest is sufficient for daily needs (%)	Dummy: 1 = yes; 0 = no/not sure/neutral
Food security and livelihoods and assets	
Meet daily needs (%)	Dummy: 1 = we can just meet our daily needs and have no extra things/we have enough to meet our daily needs and have some extra things/we can meet our daily needs and save money afterwards too; 0 = we have difficulty meeting our daily needs
Food shortages (%)	Dummy: 1 = yes; 0 = no/not sure
Food consumption score (range, 0–112)	Continuous
Low dietary diversity (%)	Dummy: 1 = consumed 4 or fewer of the food groups in the previous week; 0 = consumed more than 4 food groups
Number of crops	Continuous
Number of livestock	Continuous
Size of farm/forest land (per HH member)	Continuous
Assets index	Weighted index using data on ownership/usage of electricity, telephone, iron, refrigerator, generator, clock, bed, sofa, radio, mobile phone, and solar panel
Sources of income	Continuous
Water, sanitation, and hygiene	
Safe water source, dry season (%)	Dummy: 1 = improved source or water treated; 0 = no improved source and no treatment
Safe water source, wet season (%)	Dummy: 1 = improved source or water treated; 0 = no improved source and no treatment
Improved toilet (%)	Dummy: 1 = household has improved toilet; 0 = no improved toilet
Water and soap/ash/sand (%)	Dummy: 1 = household has handwashing place with water and cleaning agent; 0 = household has no handwashing place, or only has water ready for handwashing without cleaning agent
Adult use of mosquito bed nets (%)	Dummy: 1 = respondent of the reproductive health section slept under a net the previous night; 0 = respondent did not
Women’s highest education level	Categorical: 0 = none or less than primary; 1 = some primary; 2 = completed primary; 3 = some secondary; 4 = completed secondary; 5 = higher than secondary
Climate change awareness	
Heard of CC (%)	Dummy: 1 = yes; 0 = no/not sure
Observed changes in weather (%)	Dummy: 1 = yes; 0 = no/not sure
CC will have negative effect (%)	Dummy: 1 = negative effect; 0 = no/positive effect/not sure
Behavior change due to CC	Dummy: 1 = yes; 0 = no/not sure
Family planning and access to MCH care	
Know about FP (%)	Dummy: 1 = yes; 0 = no
Approve of FP (%)	Dummy: 1 = approve; 0 = disapprove/not sure
Used FP (%)	Dummy: 1 = yes; 0 = no
Better access to FP and health services (%)	Dummy: 1 = improved; 0 = deteriorated/no change
Unmet family planning need (%)	Dummy: 1 = unmet need; 0 = met need/no unmet need/infecund
Visited health facility (%)	Dummy: 1 = yes; 0 = no
Home health visit (%)	Dummy: 1 = yes; 0 = no
# of children	Continuous
Birth spacing (months)	Continuous
Want more children (%)	Dummy: 1 = yes; 0 = no

*FP* family planning, *MCH* maternal and child health

**Table 5:** Correlation matrix for all model variables

Variables	Mean	SD	1	2	3	4	5	6	7	8	9	10	11	12	13	14	15	16	17	18	19	20	21	22	23	24	25	26	27	28	29	30	31	32	33	34	35	36
1 HH male	83.1	37.5																																				
2 HH age	44.2	14.5	*− 0.04*																																			
3 HH employed/self employed	31.5	46.5	*0.04*	*− 0.02*																																		
4 Fishing for income	19.9	40.0	*−* 0.11	*− 0.03*	*−* 0.07																																	
5 Northern village	63.1	48.3	*0.03*	0.07	*− 0.05*	*0.03*																																
6 PHE participation	10.1	30.4	*− 0.05*	*− 0.04*	0.11	*0.04*	*0.04*																															
7 Know about FP	63.4	48.2	*−* 0.07	*−* 0.09	0.15	0.11	*− 0.05*	0.16																														
8 Approve of FP	74.5	43.6	*− 0.01*	*− 0.02*	*0.03*	*0.03*	*0.06*	*0.04*	0.16																													
9 Better access to FP and	55.9	49.7	*− 0.05*	*− 0.05*	0.14	0.07	0.07	0.11	0.27	0.12																												
10 Visited health facility	72.2	44.9	*0.00*	*− 0.05*	0.08	0.11	0.10	*− 0.01*	0.13	0.12	0.15																											
11 Home health visit	10.2	30.3	*− 0.02*	*− 0.04*	*0.00*	*0.03*	*0.01*	*0.05*	0.12	0.08	0.06	0.09																										
12 Influence on village	35.8	48.0	*−* 0.12	*0.03*	0.12	*− 0.03*	*− 0.02*	0.11	0.16	0.11	0.10	*0.02*	*0.04*																									
13 TANAPA positive relationship	40.3	49.1	*−* 0.06	*− 0.04*	*0.01*	*0.06*	0.12	0.17	0.08	0.10	0.18	0.18	0.13	0.23																								
14 Organization member	16.1	36.8	*−* 0.08	*− 0.03*	0.14	0.14	0.08	0.22	0.16	0.10	0.13	*0.05*	*0.01*	0.20	0.18																							
15 Attended public meeting	35.9	48.0	*−* 0.07	*− 0.03*	*0.03*	0.09	*0.04*	0.09	0.17	0.11	0.09	*0.07*	*0.05*	0.15	0.14	0.25																						
16 Member of BMU	12.1	32.6	*−* 0.12	*− 0.02*	0.08	0.17	*− 0.06*	0.19	0.13	0.06	0.13	0.07	0.13	0.08	0.16	0.26	0.18																					
17 Meet daily needs	60.1	48.9	*−* 0.16	*0.05*	0.09	*0.02*	*0.04*	0.07	*0.03*	0.08	0.07	*− 0.07*	*−* 0.07	*0.05*	0.13	0.07	*0.04*	*0.02*																				
18 Food shortages	42.3	49.4	*− 0.04*	0.07	0.09	*− 0.05*	*0.02*	*0.03*	*0.03*	*− 0.01*	*0.07*	*− 0.09*	*− 0.01*	0.07	0.07	0.09	*0.01*	*− 0.05*	0.25																			
19 Food Consumption Score	51.8	12.2	−0.08	*0.00*	0.12	0.08	*− 0.03*	*0.01*	0.12	*0.04*	*0.06*	*0.00*	*− 0.04*	0.07	*0.02*	0.09	0.08	0.04	0.15	0.16																		
20 Number of crops	3.6	1.9	*−* 0.08	*0.03*	*0.00*	*− 0.06*	*0.02*	*0.04*	*− 0.03*	*0.03*	*− 0.06*	*− 0.01*	*− 0.04*	*0.06*	*0.02*	*0.05*	*0.00*	*− 0.02*	0.13	0.18	0.16																	
21 Number of livestock	6.3	10.9	*−* 0.13	*0.03*	*0.04*	*− 0.01*	*−* 0.11	*0.03*	*0.05*	*− 0.03*	*0.00*	*−* 0.08	*− 0.05*	0.07	*− 0.01*	0.11	0.06	*0.03*	0.16	0.11	0.19	0.22																
22 Assets index	0.0	1.0	*−* 0.18	*− 0.03*	0.22	*− 0.03*	0.14	0.10	0.10	*0.06*	0.07	*0.07*	*0.05*	0.15	0.12	0.16	0.07	0.06	0.17	0.21	0.25	0.12	0.16															
23 Heard of CC	26.7	44.3	*−* 0.11	*0.01*	0.07	*− 0.02*	0.10	0.15	0.17	0.11	0.08	0.11	*0.00*	0.20	0.11	0.15	0.14	0.11	*0.06*	*0.00*	0.10	*0.04*	0.07	0.18														
24 Observed changes in weather	55.3	49.7	*−* 0.11	*0.00*	*0.05*	*0.01*	0.08	0.07	0.07	0.08	*0.04*	0.09	0.08	0.18	0.13	0.17	0.12	0.09	*0.00*	*0.01*	0.10	*0.05*	*− 0.01*	0.18	0.30													
25 CC will have negative effect	33.3	47.1	*− 0.06*	*− 0.03*	0.16	*0.05*	*− 0.02*	*0.03*	0.13	0.07	0.07	0.12	*0.06*	0.11	*0.03*	0.17	0.08	*0.05*	*− 0.05*	*0.00*	*0.03*	*0.02*	*0.03*	0.14	0.23	0.30												
26 Forests should be conserved	88.1	32.4	*− 0.03*	*− 0.04*	0.10	*0.06*	*0.05*	0.06	0.11	0.12	0.07	*0.04*	*0.02*	0.13	0.13	0.10	0.12	*0.03*	*0.06*	*− 0.02*	*0.06*	*− 0.02*	*0.00*	0.09	0.13	0.16	*0.05*											
27 Wildlife should be conserved	88.4	32.0	*−* 0.08	*− 0.03*	*0.06*	0.07	*0.05*	*0.06*	0.12	0.15	0.08	0.09	*0.02*	0.10	0.18	0.08	0.10	0.07	*0.06*	*− 0.02*	0.08	*− 0.02*	*− 0.02*	0.10	0.13	0.17	0.10	0.43										
28 National park should be conserved	68.3	46.5	*−* 0.07	*− 0.03*	0.08	0.10	0.13	0.13	0.16	0.16	0.10	0.14	*0.02*	0.15	0.17	0.13	0.14	*0.04*	*0.05*	*0.02*	0.07	*0.03*	*0.05*	0.10	0.12	0.14	0.09	0.35	0.54									
29 Deforestation causes siltation	68.3	46.5	*−* 0.07	*− 0.03*	0.08	0.10	0.13	0.13	0.16	0.16	0.10	0.14	*0.02*	0.15	0.17	0.13	0.14	*0.04*	*0.05*	*− 0.02*	0.09	*0.03*	*− 0.01*	0.13	0.20	0.16	0.10	0.28	0.28	0.27								
30 Siltation harms fish	35.5	47.9	*− 0.04*	*0.00*	*0.04*	*0.03*	*0.06*	0.12	0.07	0.08	0.08	*0.04*	*0.04*	0.14	0.18	0.16	0.14	0.09	*0.05*	*0.05*	0.08	*− 0.01*	*− 0.03*	0.07	0.17	0.17	*0.06*	0.16	0.20	0.22	0.42							
31 National park benefits	54.2	50.0	*− 0.01*	*0.04*	*0.02*	*0.03*	0.09	0.10	*0.06*	0.12	0.12	*0.01*	0.10	0.12	0.34	0.14	0.10	0.12	0.11	0.08	0.07	*0.05*	*− 0.01*	0.07	0.12	0.18	*0.01*	0.21	0.26	0.25	0.20	0.30						
32 Safe water source, dry season	67.6	46.8	*0.04*	*0.00*	*0.00*	*0.01*	0.20	*0.04*	*0.02*	*0.01*	0.12	0.10	*− 0.03*	*0.02*	*0.05*	*0.01*	*0.01*	*− 0.03*	*0.04*	*0.01*	0.06	*0.00*	*−* 0.09	0.11	*0.05*	*− 0.03*	*− 0.01*	0.07	0.07	*0.06*	0.07	0.09	*0.06*					
33 Improved toilet	18.8	39.1	*− 0.02*	*0.02*	*0.06*	*0.06*	0.11	0.11	0.07	*0.03*	*0.03*	*0.06*	*0.06*	*0.06*	0.10	*0.04*	*0.03*	*0.02*	*0.01*	*0.05*	*0.05*	*0.00*	*0.02*	0.18	*0.06*	0.10	0.08	0.14	0.10	0.08	0.09	*0.06*	*− 0.02*	0.14				
34 Water and soap/ash/sand	46.4	49.9	*0.01*	*0.03*	0.15	0.07	*0.03*	0.10	0.09	*0.04*	0.09	0.08	*0.06*	0.07	0.08	0.07	0.08	*0.04*	*− 0.01*	*− 0.01*	0.10	*0.02*	*0.01*	0.20	0.09	*0.02*	0.16	*0.06*	0.08	*0.04*	0.09	*0.03*	*0.00*	0.15	0.14			
35 Behavior change due to CC	12.8	33.4	*−* 0.13	*− 0.03*	0.09	0.09	*0.02*	0.09	0.10	0.09	*0.02*	*0.04*	*0.04*	0.13	0.13	0.15	0.10	0.14	0.08	*0.00*	0.12	0.09	*0.06*	*0.04*	0.16	*0.02*	*0.07*	*0.06*	*0.06*	*0.02*	0.09	*0.05*	*0.07*	*0.06*	*0.04*	0.10		
36 Use mosquito net	88.9	31.5	31.5	*0.02*	*0.03*	*0.03*	0.10	*0.06*	*0.06*	0.09	0.11	0.16	0.09	0.07	*0.07*	*0.07*	*0.05*	*0.05*	*−0.04*	*0.04*	0.10	*0.06*	*− 0.07*	*0.05*	0.07	*0.05*	*0.01*	0.09	*0.06*	*0.05*	0.08	*0.03*	*0.05*	0.13	0.08	0.09	*0.08*	
37 Women’s education	2.4	1.1	*− 0.04*	*− 0.06*	0.09	*0.04*	0.07	0.09	0.10	0.09	0.10	0.08	*0.03*	0.07	0.09	0.11	0.08	0.09	0.08	*0.04*	0.11	*0.05*	0.07	0.19	0.14	*0.06*	*0.05*	0.16	0.12	0.15	0.16	*0.06*	0.11	0.13	0.09	0.11	*0.04*	*0.04*

Correlations that are italicized were not significant at *p* < 0.05

*FP* family planning, *CC* climate change

**Table 6:** First-order measurement model covariates

	Unstandardized coefficient (SE)	Standardized coefficient
Social cohesion–participation (SC–P)		
Household (HH) head age	0.00 (0.00)	0.02
HH head female	*−* 0.20** (0.07)	*−* 0.18
HH size	0.01 (0.01)	0.03
Anyone in HH employed/self-employed	0.01 (0.05)	0.01
Anyone in HH fishes	0.02 (0.06)	0.02
Model household	0.33*** (0.09)	0.17
Village in north	0.04 (0.05)	0.04
Natural resource protection attitudes (NR)		
HH head age	0.00 (0.00)	0.02
HH head female	*−* 0.09* (0.08)	*−* 0.05
HH size	*−* 0.01 (0.01)	*−* 0.04
Anyone in HH employed/self-employed	*−* 0.03 (0.07)	*−* 0.02
Anyone in HH fishes	0.06 (0.09)	0.03
Model household	0.22 (0.12)	0.09
Village in north	0.13* (0.07)	0.08
Food Security and livelihoods and assets (FLA)		
HH head age	0.00 (0.00)	0.05
HH head female	*−* 0.29*** (0.07)	*−* 0.23
HH size	0.04*** (0.01)	0.23
Anyone in HH employed/self-employed	0.21*** (0.05)	0.20
Anyone in HH fishes	*−* 0.09 (0.05)	*−* 0.07
Model household	0.05 (0.07)	0.03
Village in north	0.02 (0.04)	0.02
Water, sanitation, and hygiene (WASH)		
HH head age	0.00 (0.00)	0.08
HH head female	0.08 (0.07)	0.06
HH size	*−* 0.01 (0.01)	*−* 0.02
Anyone in HH employed/self-employed	0.12* (0.06)	0.11
Anyone in HH fishes	0.05 (0.07)	0.04
Model household	0.19* (0.09)	0.11
Village in north	0.23*** (0.06)	0.23
Climate change awareness (CC-A)		
HH head age	0.00 (0.00)	0.04
HH head female	*−* 0.33*** (0.10)	*−* 0.19
HH size	0.00 (0.01)	0.01
Anyone in HH employed/self-employed	0.08 (0.08)	0.05
Anyone in HH fishes	*−* 0.16 (0.09)	*−* 0.08
Model household	0.11 (0.12)	0.04
Village in north	0.12 (0.08)	0.07
Family planning and access to MCH care (FP-MCH)		
HH head age	*−* 0.01** (0.00)	*−* 0.13
HH head female	*−* 0.15 (0.08)	*−* 0.08
HH size	0.02* (0.01)	0.10
Anyone in HH employed/self-employed	0.37*** (0.07)	0.26
Anyone in HH fishes	0.33*** (0.08)	0.20
Model household	0.42*** (0.10)	0.23
Village in north	0.16** (0.06)	0.12

*N* = 1010

****p* < 0.001; ***p* < 0.01; **p* < 0.05
